# A multitask deep learning radiomics model for predicting the macrotrabecular-massive subtype and prognosis of hepatocellular carcinoma after hepatic arterial infusion chemotherapy

**DOI:** 10.1007/s11547-023-01719-1

**Published:** 2023-10-06

**Authors:** Xuelei He, Kai Li, Ran Wei, Mengxuan Zuo, Wang Yao, Zechen Zheng, Xiaowei He, Yan Fu, Chengzhi Li, Chao An, Wendao Liu

**Affiliations:** 1 Department of Interventional Therapy, Guangdong Provincial Hospital of Chinese, Medicine and Guangdong Provincial Academy of Chinese Medical Sciences, No. 111 Dade Road, Guangzhou, 510080 Guangdong People’s Republic of China; 2 School of Information Sciences and Technology, Northwest University, Xi’an, 710127 Shaanxi Province People’s Republic of China; 3 Department of Minimal Invasive Intervention, Sun Yat-sen University Cancer Center; State Key Laboratory of Oncology in South China, Collaborative Innovation Center for Cancer Medicine, 651, Dongfeng East Road, Guangzhou, 510060 People’s Republic of China; 4 Department of Ultrasound, The Third Affiliated Hospital of Sun Yat-sen University, Guangzhou, 510080 Province Guangdong People’s Republic of China; 5 Department of Interventional Radiology and Vascular Surgery, The Third Affiliated Hospital of Sun Yat-sen University, Guangzhou, 510080 Province Guangdong People’s Republic of China; 6 Department of Interventional Radiology and Vascular Surgery, The First Affiliated Hospital of Jinan University, Guangzhou, 510060 People’s Republic of China; 7 Department of Interventional Therapy, National Cancer Center/National Clinical, Research Center for Cancer/Cancer Hospital, Chinese Academy of Medical, Sciences and Peking Union Medical College, Beijing, 100021 People’s Republic of China

**Keywords:** Hepatocellular carcinoma, Deep learning, Hepatic arterial infusion chemotherapy, Macrotrabecular massive, Prognostic risk stratification

## Abstract

**Background:**

The macrotrabecular-massive (MTM) is a special subtype of hepatocellular carcinoma (HCC), which has commonly a dismal prognosis. This study aimed to develop a multitask deep learning radiomics (MDLR) model for predicting MTM and HCC patients’ prognosis after hepatic arterial infusion chemotherapy (HAIC).

**Methods:**

From June 2018 to March 2020, 158 eligible patients with HCC who underwent surgery were retrospectively enrolled in MTM related cohorts, and 752 HCC patients who underwent HAIC were included in HAIC related cohorts during the same period. DLR features were extracted from dual-phase (arterial phase and venous phase) contrast-enhanced computed tomography (CECT) of the entire liver region. Then, an MDLR model was used for the simultaneous prediction of the MTM subtype and patient prognosis after HAIC. The MDLR model for prognostic risk stratification incorporated DLR signatures, clinical variables and MTM subtype.

**Findings:**

The predictive performance of the DLR model for the MTM subtype was 0.968 in the training cohort [TC], 0.912 in the internal test cohort [ITC] and 0.773 in the external test cohort [ETC], respectively. Multivariable analysis identified portal vein tumor thrombus (PVTT) (*p* = 0.012), HAIC response (*p* < 0.001), HAIC sessions (*p* < 0.001) and MTM subtype (*p* < 0.001) as indicators of poor prognosis. After incorporating DLR signatures, the MDLR model yielded the best performance among all models (AUC, 0.855 in the TC, 0.805 in the ITC and 0.792 in the ETC). With these variables, the MDLR model provided two risk strata for overall survival (OS) in the TC: low risk (5-year OS, 44.9%) and high risk (5-year OS, 4.9%).

**Interpretation:**

A tool based on MDLR was developed to consider that the MTM is an important prognosis factor for HCC patients. MDLR showed outstanding performance for the prognostic risk stratification of HCC patients who underwent HAIC and may help physicians with therapeutic decision making and surveillance strategy selection in clinical practice.

**Supplementary Information:**

The online version contains supplementary material available at 10.1007/s11547-023-01719-1.

## Introduction

Hepatocellular carcinoma (HCC) is the fourth most common malignant tumour and ranks as the second leading cause of cancer death globally [1]. Unfortunately, > 70% of patients with HCC often have a high tumour burden when they receive the initial diagnosis [2]. Hepatic arterial infusion chemotherapy (HAIC) is a promising option for large HCC that provides sustained local high concentrations of chemotherapy agents in the tumour [3]. It easier to obtain a high objective response rate (ORR) for large HCC with multicycle HAIC, which can enable further conversion therapy. Shi Ming et al. showed that HAIC with the FOLFOX regimen (oxaliplatin plus fluorouracil and leucovorin) yielded a better median overall survival (OS, 23.1 months) and ORR (48%) than transarterial chemoembolization (TACE) for large HCC (largest diameter > 7 cm) in a randomized phase III trial [4]. Moreover, immunotherapies and and multitargeted tyrosine kinase inhibitors (TKIs) including sorafenib and lenvatinib have present outstanding ORR and survival benefit for advanced HCC [5, 6].

The macrotrabecular-massive (MTM) subtype, as an amorphologic HCC variant with angiogenesis, has been reported to have a dismal prognosis in previous reports [7, 8]. Patients with this subtype of HCC should be specifically diagnosed before surgery, but histopathologic examinations remain lacking. A series of studies have identified intratumor necrosis or ischemia as an independent predictor of the MTM subtype. And MTM subtype could be effectively diagnosed by these features combined with intratumor fat deficiency. Moreover, compared with non-MTM-HCC, several research found that MTM-HCC was often larger with more prone to intratumor necrosis and frequently exhibit irregular rim-like arterial phase enhancement (IRE) with a stronger invasion ability [9–11]. Although the abovementioned MRI features could achieve high accuracy for predicting the MTM subtype in previous studies, potential selection bias resulting from interobserver variation was difficult to avoid. Over the past decade, an increasing number of quantitative and qualitative image analysis methods for the prediction of the MTM subtype have been proposed in oncological practice. For example, radiomics converts images into quantitative data in a high-throughput manner, making it a feasible and precise approach for outcome prediction. However, these analyses require the formulation of predefined criteria and manual or semiautomatic segmentation of the region of interest (i.e., the tumour and margin region) [12, 13]. However, deep learning (DL), as a data-driven approach, has been increasingly applied towards automatic design and organization based on the predictive ability of specific features instead of human performance [14, 15].

Therefore, further studies are required to support the robustness and accuracy of the DL radiomics (DLR) approach for predicting the MTM subtype and patient prognosis. The aim of our study was to develop and validate a multitask DLR-based model based on preoperative CT for predicting the MTM subtype and prognosis of HCC patients who underwent HAIC based on multimodal data integrating clinical variables, DLR score and MTM subtype.

## Materials and methods

This retrospective, multi-institutional study protocol obtained approval from the Institutional Review Board of all participating hospitals and was conducted following the principles of the 1975 Helsinki Declaration. Due to the retrospective nature of this study, the requirement for written informed consent was waived.

### Patient enrolment

All HCC patients were diagnosed based on the European Association for the Study of Liver (EASL) and the American Association for the Study of Liver Disease (AASLD) guidelines [16, 17]. Between June 2018 and March 2020, a total of 159 consecutive patients with large HCC who received surgical resection (SR) were reviewed and underwent a standard contrast-enhanced computed tomography (CECT) examination within 2 weeks before SR in a tertiary high-volume hospital. The histologic examination of tumour specimens was performed by two pathologists (reader 1, L.L., and reader 2, P.W., with 10 years of experience) by serially examining multiple pathologic specimens. The intraclass correlation coefficient (ICC) was calculated as the metric for reproducibility evaluation. Pathologic features with both intra- and interobserver ICCs higher than 0.9 were selected. The MTM subtype was defined as *a* > 50% macrotrabecular architectural pattern present after haematoxylin–eosin staining.

**Fig. 1:**
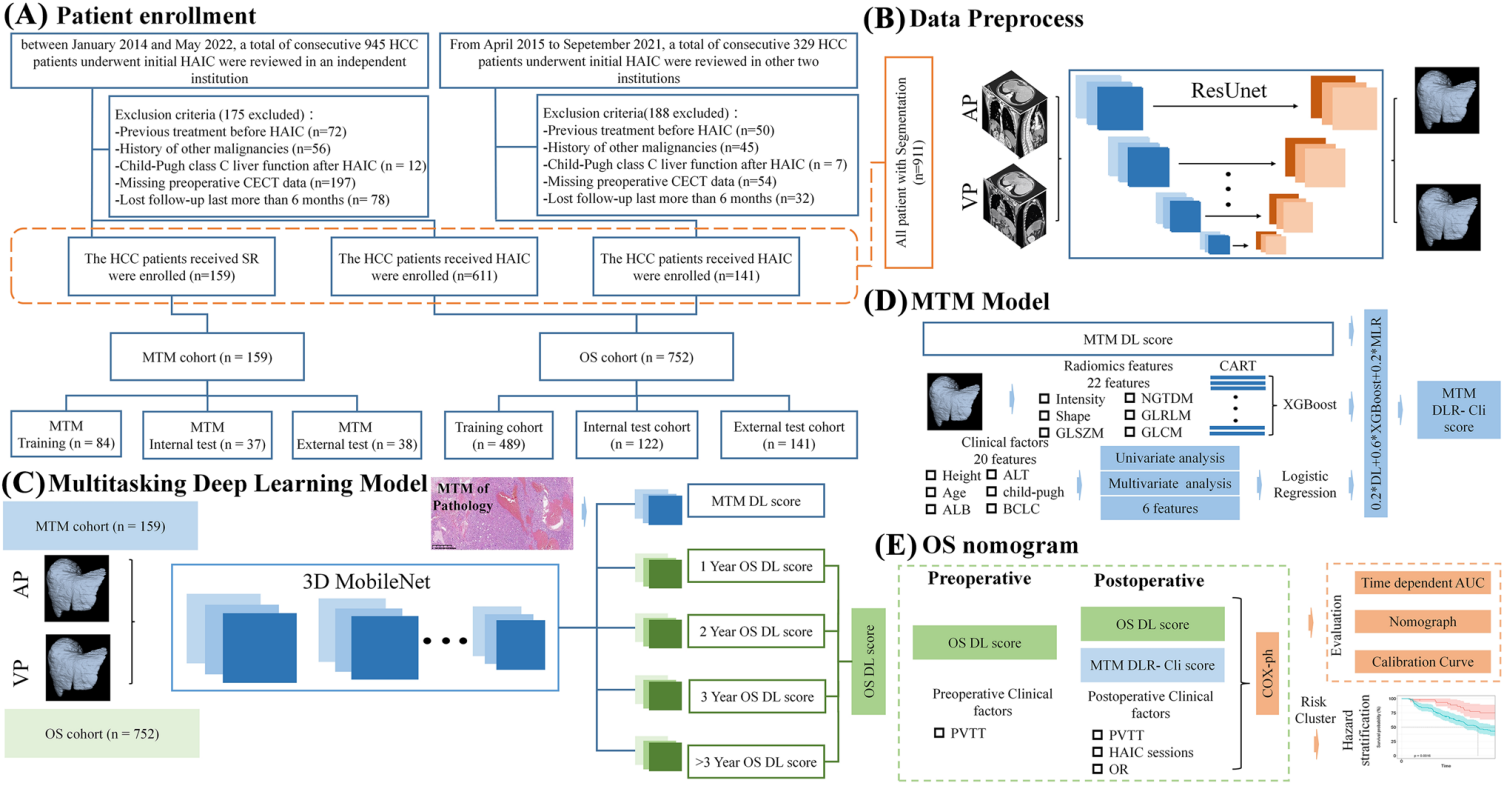
Flowcharts show HCC patient recruitment process and MDLR model construction. **A** HCC patient recruitment. **B** Data preprocess. **C** MTM model. **D** OS nomogram; **E** MDLR model construction. MTM = macrotrabecular-massive HCC = hepatocellular carcinoma (HCC), MDLR = multitask deep learning radiomics OS = overall surviva

Another cohort consisted of 1367 patients with HCC who received initial HAIC as the first-line therapy between January 2014 and May 2022. Figure 1A demonstrates the enrolment pathways of HCC patients who underwent HAIC. The inclusion criteria were as follows: (a) age 18–75 years; (b) Eastern Cooperative Oncology Group (ECOG) performance status < 2; (c) Child‒Pugh class A or B liver function; and (d) management of HAIC with the FOLFOX regimen (FOLFOX-HAIC). The exclusion criteria were as follows: (a) any treatment before HAIC; (b) HCC combined with other malignancies; (c) a maximum tumour diameter ≤ 5 cm; (d) simultaneous treatment of TACE combined with HAIC; and (e) loss to follow-up after > 6 months. The reasons for using HAIC rather than surgery or systematic chemotherapy, the HAIC procedures, and criteria for protocol discontinuation are shown in supplementary information E1.1–1.3. Moreover, the preoperative CECT scan protocol is described in supplementary information E1.4.

### Follow-up protocol and endpoints

In this study, enrolled patients were censored at the last follow-up (October 30, 2022). After a thorough HAIC protocol was completed, the serum alpha-fetoprotein (AFP) levels and contrast-enhanced CT or MRI were repeated in 3–6-month intervals, at approximately 3-month intervals in the first year and 6-month intervals thereafter. The responses to HAIC were assessed by dynamic contrast-enhanced images acquired before and after HAIC. The assessment was performed independently every 4–6 weeks after initial HAIC by two radiologists (reader 1, L.Z.L., and reader 2, J.Z., with 10 years of experience) who were blinded to the HAIC procedure at the time of data collection. According to the modified Response Evaluation Criteria in Solid Tumor (mRECIST), the responses were divided into complete response (CR), partial response (PR), stable disease (SD), and progressive disease (PD) [18]. The primary endpoint was OS, which was calculated from the date of initial treatment to the date of death from any cause or date of last follow-up. Thirty-four clinical variables were collected for analysis as predictors of HAIC prognosis and are listed in supplementary information E 1.5.

### Study design

In this study, we used dual-phase (arterial phase and venous phase) CECT data collected from MTM related cohort comprising 159 patients who received SR and HAIC related cohort comprising 752 patients who received HAIC to develop and validate a multitask deep learning radiomic nomogram (MDLRN). Figure 1B–E shows the MDLRM pipeline, including the image segmentation of regions of interest (ROIs), feature extraction and selection, signature building, and model construction. The detailed automatic delineation was listed in supplementary information E 1.6. We used the clinical and CECT data from one tertiary high-volume institution as the training cohort (TC, *n* = 459) and internal testing cohort (ITC, *n* = 122) and the clinical data from 4 medical centres as the external test cohort (ETC, *n* = 141).

### MTM-related score

The first step was a histologic-related ML scoring model for prediction of MTM status. A 3D MobileNetV1 Structure (shown in sTable 1 and sFigure 1) Deep Learning Model for prediction of MTM status was constructed to extract the High-Level Image features. Then 22 radiomics features were selected and constructed the model by XGBoost with CART base-classification [19]. There were also 6 clinical factors selected to construct the clinical model. At last, the score of three models (Clinical, Deep Learning and Radiomics, DLR-Cli) were added with weight to build a MTM-related score model. The detailed information of multi-task deep learning (MDL) model construction, MTM radiomic model construction and procedure of DLR-Cli model construction were described in supplementary methods E1.8–1.9.

### Prognostic score for survival after HAIC

In the next step, we built and validated a MDLRN integrated a MTM related biomarker, DL score extracted from tumor and other clinical information for prediction of OS in the 752 patients in OS cohorts. The same 3D MobileNetV1 multi-task model was used for multi-task OS predictions, which was described in supplementary information E1.6. The predicted MTM-score, DL-score, pre- and post-operative clinical variables were then integrated into a new Cox-PH model to obtain a precise estimation of the survival time of an individual patient received HAIC.

### Statistical analysis

Statistical analysis was performed using the survival and rms packages of R software version 3.6.3 (http://www.r-project.org/). Continuous variables were presented as mean ± standard deviation (SD) or median with interquartile range (IQR) and compared using the Kruskal–Wallis test, while categorical variables were presented as frequencies with percentages and compared using the chi-squared test. Univariable and multivariable logistic regression analyses were applied to calculate the hazard ratios (HRs) and corresponding 95% confidence intervals (CIs) of variables and identify independent significant risk factors. The OS curves of different subgroups were compared using the Kaplan–Meier method with the log-rank test, and the AUCs of different models were compared by the DeLong test. The predictive parameters, including accuracy, sensitivity, specificity, positive predictive value (PPV), and negative predictive value (NPV), were also calculated to assess model performance.

All tests of significance were two-sided, and a *p* value < 0.05 was interpreted to carry statistical significance.

## Results

### Baseline characteristics

In MTM related cohort, 74.2% (118/159) of patients were diagnosed pathologically with the MTM subtype. The baseline characteristics stratified by MTM status are shown in Table 1**.** Among all variables, age < 65 years and Edmondson-Steiner grade III–IV were found to be more prevalent in the MTM group than in the non-MTM group (*p* = 0.032, < 0.001). Other variables showed a similar distribution between the two groups. In HAIC related cohorts, a total of 752 treatment-naïve patients with HCC (80 females and 672 males; mean age, 54.2 ± 11.8 years) met the inclusion criteria. The clinicopathologic characteristics of the HCC patients who underwent HAIC in the three cohorts are outlined in Table 2. At the final follow-up, the mortality rates were 61.1% (299/489) in the TC, 71.3% (87/122) in the VC, and 35.5% (50/141) in the EVC. The baseline characteristics of the abovementioned two cohorts are shown in sTable 2.

**Table 1:** Patient characteristics according to the MTM subtype

Variables	MTM (*n* = 118)	Non-MTM (*n* = 41)	*p*-value
Age (years)			0.075
≤ 65	12 (10.17%)	0 (0.00%)	
> 65	106 (89.83%)	41 (100%)	
Sex			0.507
Female	16 (13.56%)	8 (19.51%)	
Male	102 (86.44%)	33 (80.49%)	
HBV			1.00
Absence	5 (4.24%)	2 (4.88%)	
Presence	113 (95.76%)	39 (95.12%)	
HCC number			0.837
1	56 (47.46%)	22 (53.66%)	
2	5 (4.24%)	2 (4.88%)	
3	1 (0.84%)	0 (0.00%)	
> 3	56 (47.46%)	17 (41.46%)	
HCC diameter, cm			0.146
≤ 7	11 (10.28%)	8 (19.51%)	
> 7	107 (89.72%)	33 (80.49%)	
ALBI grade			0.319
1	6 (5.08%)	0 (0.00%)	
2–3	112 (94.92%)	41 (100%)	
AFP, ng/ml			0.642
≤ 400	45 (38.14%)	18 (43.90%)	
> 400	73 (61.86%)	23 (56.10%)	
BCLC stage			0.915
A and B	43 (36.45%)	16 (39.02%)	
C	75 (63.55%)	25 (60.98%)	

Data are number of patients; data in parentheses are percentage of patients unless otherwise indicated. The data in two groups were compared by using the Chi square test

*HAIC HBV* viral hepatitis type B, *AFP* α-fetoprotein

**Table 2:** Baseline characteristics of patients with large HCC who received HAIC of FOLFOX

Variables	Training cohort (*n* = 489)		*p* value	Internal validation cohort (*n* = 122)		*p* value	External validation cohort (*n* = 141)		*p* value
	Survial (*n* = 190)	Death (*n* = 299)		Survial (*n* = 35)	Death (*n* = 87)		Survial (*n* = 50)	Death (*n* = 91)	
*Demographics*									
Age (years), mean ± SD	51.2 ± 12.1	51.3 ± 11.9	0.718	51.2 ± 10.6	50.4 ± 11.6	0.478	51.4 ± 11.6	52.8 ± 10.6	0.019
Gender			0.007			1.00			1.00
Female	11 (5.79%)	42 (14.05%)		4 (11.43%)	10 (11.49%)		5 (10.00%)	8 (8.79%)	
Male	179 (94.21%)	257 (85.95%)		31 (88.57%)	77 (88.51%)		45 (90.00%)	83 (91.21%)	
BMI, mean ± SD	21.63 ± 2.76	21.80 ± 2.41	0.806	21.65 ± 3.50	21.22 ± 2.19	0.935	21.82 ± 1.76	21.55 ± 2.57	0.726
PS			0.350			0.692			0.975
ECOG 0	178 (93.68%)	287 (95.99%)		33 (94.29%)	85 (97.70%)		47 (94.00%)	84 (92.31%)	
ECOG 1	12 (6.32%)	12 (4.01%)		2 (5.71%)	2 (2.30%)		3 (6.00%)	7 (7.69%)	
Comorbidities			0.154			0.257			0.095
Absence	162 (85.26%)	269 (89.97%)		28 (80.00%)	78 (89.66%)		39 (78.00%)	75 (82.42%)	
Presence	28 (14.74%)	30 (10.03%)		7 (20.00%)	9 (10.34%)		11 (22.00%)	16 (17.58%)	
HBV			0.020			0.788			0.647
Absence	22 (11.58%)	16 (5.35%)		2 (5.71%)	8 (9.20%)		7 (14.00%)	9 (9.89%)	
Presence	168 (88.42%)	283 (94.65%)		33 (94.29%)	79 (90.80%)		43 (86.00%)	82 (90.11%)	
Ascites			1.000			0.600			0.619
Absence	167 (87.89%)	262 (87.63%)		31 (88.57%)	72 (82.76%)		38 (76.00%)	73 (80.22%)	
Presence	23 (12.11%)	37 (12.37%)		4 (11.43%)	15 (17.24%)		12 (24.00%)	17 (19.78%)	
ALBI grade			0.684			0.493			1.000
1	94 (49.47%)	141 (47.16%)		15 (42.86%)	45 (51.72%)		29 (58.00%)	53 (58.24%)	
2–3	96 (50.53%)	158 (52.84%)		20 (57.14%)	42 (48.28%)		21 (42.00%)	38 (41.76%)	
Metastasis			0.003			0.314			0.312
Absence	114 (60%)	155 (51.84%)		19 (54.29%)	39 (44.83%)		36 (72.00%)	51 (56.04%)	
Presence	76 (40%)	144 (48.16%)		16 (45.71%)	48 (55.17%)		14 (28.00%)	40 (43.96%)	
LN	44 (23.16%)	114 (38.13%)		7 (20.00%)	31 (35.63%)		10 (20.00%)	23 (25.27%)	
Lung	26 (13.68%)	28 (9.36%)		6 (17.14%)	12 (13.79%)		3 (6.00%)	13 (14.29%)	
Adrenal	3 (1.58%)	1 (0.335%)		0 (0.00%)	2 (2.30%)		0 (0.00%)	2 (2.20%)	
Bone	3 (1.58%)	1 (0.335%)		3 (8.57%)	3 (3.45%)		1 (2.00%)	2 (2.20%)	
*Image characteristics*									
Tumor size (cm), mean ± SD	11.10 ± 3.59	10.61 ± 3.48	0.140	12.48 ± 3.40	12.36 ± 3.17	0.825	10.56 ± 3.33	11.11 ± 3.35	0.555
No. of tumors			0.192			0.118			< 0.001
1	52 (27.37%)	71 (23.75%)		15 (42.86%)	22 (25.28%)		11 (22.00%)	43 (47.25%)	
2	15 (7.89%)	16 (5.35%)		3 (8.57%)	3 (3.45%)		2 (4.00%)	3 (3.30%)	
3	4 (2.11%)	2 (0.67%)		1 (2.86%)	3 (3.45%)		0 (0.00%)	8 (8.79%)	
> 3	119 (62.63%)	210 (70.23%)		16 (45.71%)	59 (67.82%)		37 (74.00%)	37 (40.66%)	
AFP (ng/ml)			0.451			0.626			0.119
< 400	72 (37.89%)	102 (34.11%)		14 (40.00%)	29 (33.33%)		14 (28.00%)	39 (42.86%)	
≥ 400	118 (62.11%)	197 (65.89%)		21 (60.00%)	58 (66.67%)		36 (72.00%)	52 (57.14%)	
Median AST (U/L)	66.75	72.2	0.626	96.2	80.7	0.273	76.0	69.0	0.119
Median ALT (U/L)	47.95	49.75	0.877	51.1	46.2	0.116	50.0	49.0	0.792
Median TBIL (μmol/L)	15.4	15.4	0.057	15.4	16.4	0.036	16.30	15.1	0.918
ALB (g/L), mean ± SD	39.83 ± 4.36	39.62 ± 4.33	0.612	38.19 ± 4.16	39.54 ± 4.72	0.141	40.20 ± 4.72	40.58 ± 4.80	0.290
INR, mean ± SD	1.09 ± 0.12	1.09 ± 0.11	0.951	1.13 ± 0.11	1.10 ± 0.10	0.122	1.11 ± 0.11	1.11 ± 0.13	0.890
PT (s), mean ± SD	12.39 ± 1.42	12.41 ± 1.15	0.825	12.96 ± 1.28	12.59 ± 1.16	0.126	12.68 ± 1.26	12.72 ± 1.38	0.717
Median PLT (× 10^9^)	254	229.0	0.053	232.0	263	0.190	228.5	199.0	0.452
Cre (U/L), mean ± SD	69.81 ± 15.07	68.34 ± 15.20	0.297	67.51 ± 16.59	69.07 ± 26.82	0.416	75.11 ± 13.43	78.79 ± 76.27	0.790
Median CRP (U/L)	15.77	14.75	0.052	13.55	21.93	0.358	14.05	13.17	0.103
Neu (μmol/L), mean ± SD	5.09 ± 2.35	4.81 ± 2.11	0.182	4.68 ± 2.09	5.24 ± 2.39	0.224	5.51 ± 3.96	4.67 ± 1.68	0.087
Ly (μmol/L), mean ± SD	1.49 ± 0.58	1.50 ± 0.60	0.861	1.52 ± 0.44	1.50 ± 0.53	0.890	1.51 ± 0.55	1.56 ± 0.49	0.910
*Treatment and follow-up*									
Rounds of HAIC			< 0.001			0.474			0.230
1	13 (6.84%)	62 (20.74%)		5 (14.29%)	23 (26.44%)		11 (22.00%)	14 (15.38%)	
2	35 (18.42%)	74 (24.75%)		7 (20.00%)	18 (20.69%)		13 (26.00%)	38 (41.56%)	
3	34 (17.89%)	32 (10.70%)		5 (14.29%)	12 (13.79%)		5 (10.00%)	11 (12.09%)	
> 3	108 (56.85%)	131 (43.81%)		18 (51.42%)	34 (39.08%)		21 (42.00%)	28 (30.74%)	
Sequential treatment			< 0.001			< 0.001			< 0.001
None	50 (26.32%)	209 (69.90%)		27 (77.14%)	84 (96.55%)		38 (76.00%)	84 (92.31%)	
Surgery	7 (3.68%)	0 (0.00%)		0 (0.00%)	0 (0.00%)		7 (14.00%)	1 (1.10%)	
Ablation	19 (10.00%)	6 (2.01%)		3 (8.57%)	1 (1.15%)		8 (16.00%)	1 (1.10%)	
SBRT	23 (12.11%)	18 (6.02%)		5 (14.29%)	1 (1.15%)		1 (2.00%)	3 (3.30%)	
PD-1	99 (52.11%)	30 (10.03%)		12 (34.29%)	4 (4.60%)		12 (24.00%)	10 (10.99%)	
TKI	108 (56.84%)	68 (22.74%)		16 (45.71%)	15 (17.24%)		16 (32.00%)	18 (19.78%)	
Median follow-up, months	22.5	23.7	0.563	19.8	20.5	0.778	17.8	16.3	0.578

Data are number of patients; data in parentheses are percentage of patients unless otherwise indicated. The data in two groups were compared by using the Chi square test. Non-normally distributed data is represented by median and quartile. *p *value < 0.05 suggest statistically significant differences between three cohorts

*HAIC* hepatic arterial infusion chemotherapy, *FOLFOX* oxaliplatin plus fluorouracil and leucovorin, *OR* objective responds, *SD* standard deviation, *BMI* body mass index, *PS* performance status, *ECOG* Eastern Cooperative Oncology Group, *HBV* viral hepatitis type B, *AFP* α-fetoprotein, *ALBI* albumin-bilirubin, *ALB* albumin, *ALT* alanine aminotransferase, *AST* aspartate aminotransferase, *PT* prothrombin time, *INR* international normalized ratio, *TBIL* total bilirubin, *PLT* platelet, *SBRT* stereotactic body radiation therapy, *TKI* tyrosine kinase inhibitor

### Hand-crafted radiomic and DL feature analysis

Based on the segmented liver images, a total of 5610 pre-defined radiomic features and 4132 DL features were extracted from each phase of CECT. After feature selection, 10 in AP and 12 in PP were selected as significant pre-defined radiomic features. Among all ML classifiers, XGBoost outperformed other 3 classifiers and was selected to build radiomics scores. Most of the selected pre-defined radiomic features were GLCM features, which might be related to the heterogeneity of HCC. Besides, all DL features in AP and PP were chosen to build DL scores for further analysis. Prognostic performance comparison between various of models and staging system was shown in Table 3.

**Table 3:** Prognostic Performance of DL-based models compared with staging systems after HAIC of HCC

Models	Training cohort		Internal validation cohort		External validation cohort	
	AUC	*p*-value	AUC	*p*-value	AUC	*p*-value
Preoperative nomogram	0.7099	Reference	0.7029	Reference	0.6484	Reference
Postoperative nomogram	0.8553	Reference	0.8049	Reference	0.7921	Reference
Preoperative clinical model	0.5257	< 0.001*	0.5197	< 0.001*	0.5234	< 0.001*
Postoperative clinical model	0.8136	< 0.001†	0.7548	< 0.001†	0.7546	< 0.001†
AJCC TNM	0.5274	0.002†	0.5339	0.002†	0.6360	0.003†
BCLC stage	0.5414	0.001*	0.5546	0.001*	0.6433	0.001*
CLIP classification	0.4803	0.088*	0.5234	0.002*	0.6651	0.001*
HKLC stage	0.5253	0.002*	0.6320	0.002*	0.6517	0.002*

Numbers in parentheses are the 95% confidence interval. All *p* values were obtained from analyses comparing the AUC of various models by using the Delong test

*AJCC* American Joint Committee on Cancer, *AUC* area under the receiver operating characteristic curve, *BCLC* Barcelona Clinic Liver Cancer, *CLIP* Cancer of the Liver Italian Program, *HKLC* Hong Kong Liver Cancer

*p value versus preoperative nomogram

†*p* value versus postoperative nomogram

### MTM-related score

The baseline characteristics of patients with MTM subtype were listed in sTable 3. In the TC, the deep learning radiomics (DLR) risk score was lower in the MTM group than in the non-MTM group (mean, 0.834 ± 0.097 vs. 0.177 ± 0.089; *p* < 0.001). Multivariable analysis showed that an AFP level > 400 ng/ml and the DLR risk score were independent indicators for the MTM subtype. The comparison of predictive performance among four different models (clinical, radiomics, DLR, and DLR-Cli) in three cohorts and the AUC, SENS, SPEC, PPV, and NPV data of each model are shown in sTable 4. Among all models, the DLR-Cli model showed optimal discrimination, achieving AUCs of 0.967 in the TC, 0.912 in the IVC and 0.773 in the EVC, respectively. The results of the DeLong test indicated a significant difference in performance between the clinical model and the DLR-Cli model (*p* < 0.001 in TC, *p* < 0.001 in IVC and *p* < 0.001 in EVC).

### The development and validation of the MDLRN

**Fig. 2:**
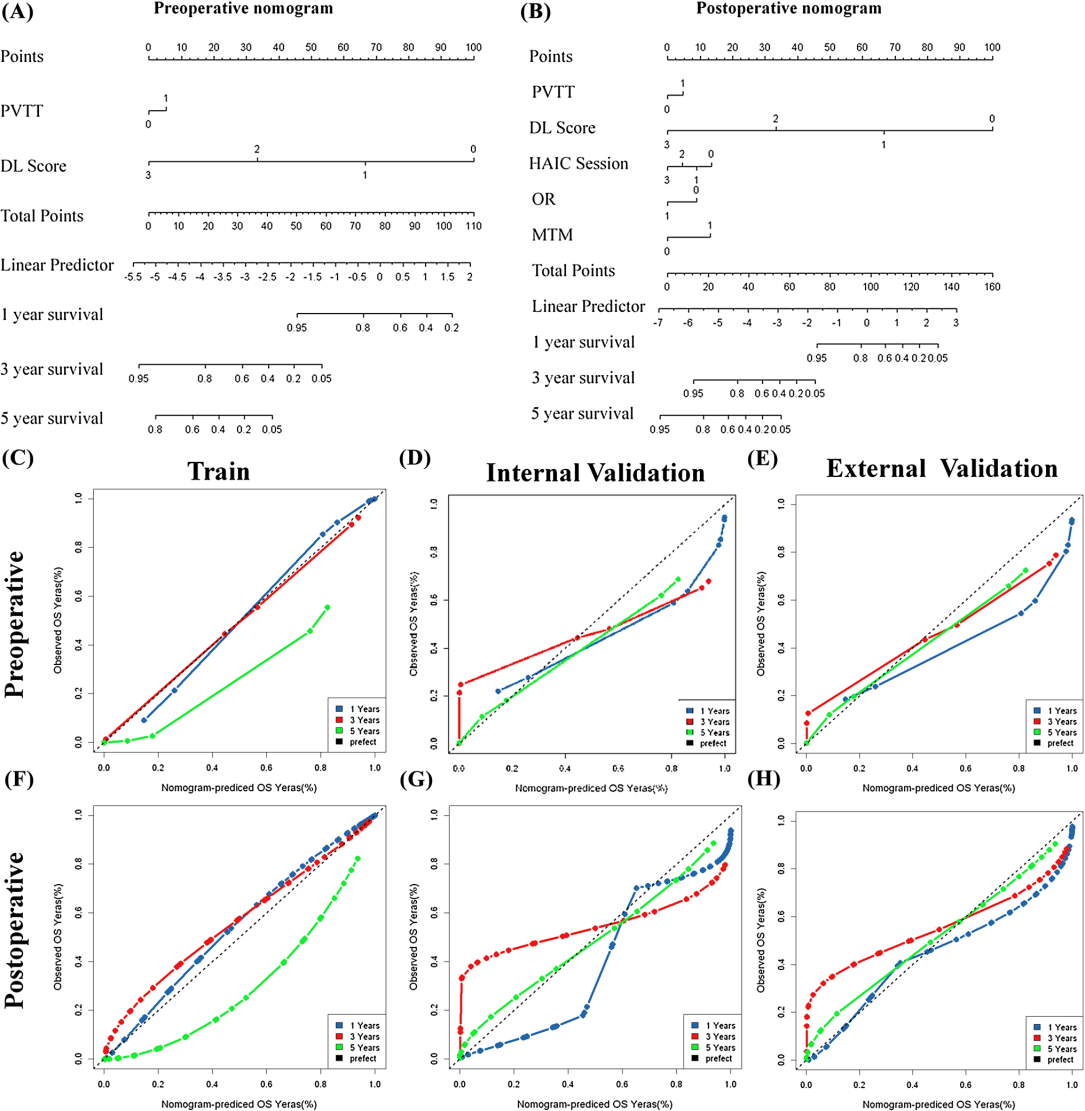
Development of prognostic nomogram for OS. **A** The pre-nomogram was established using diagnostic factors for patients who had not received HAIC treatment and had preoperative HAIC data. **B** The post-nomogram was established using multiple factors for patients who had undergone HAIC treatment and had both pre- and post -HAIC data. **C**–**E** calibration curves plotted with 1-, 3- and 5-year overall survival (OS) were well matched with the idealized 45° line for the pre-nomogram in training cohort, internal testing cohort and external testing cohort. **F**–**H** calibration curves plotted with 1-, 3- and 5-year OS were well matched with the idealized 45°line for the post-nomogram in training cohort, internal testing cohort and external testing cohort

Multivariate analysis showed that preoperative parameters, including PVTT (HR, 1.42) and DLR risk score (HR, 0.11), and postoperative parameters, including OR, HAIC sessions and MTM score, were independent risk factors for poor OS (sTable 5). The detailed performance of MDL for OS listed in sTable 6. These independently associated risk factors were used to develop the MDLRN (Fig. 2A, B), described by the formula: HR = 1.38 × PVTT + 0.54 × OR + 0.74 × HAIC sessions + 2.44 × MTM + 0.10 × DLR score. For each tumour grade, a higher total point value indicated a worse OS. The bootstrapped calibration curves plotted with 1-, 3- and 5-year OS were well matched with the idealized 45° line for the MDLRN in the three cohorts (Fig. 2C–H). To add clinical convenience, a user-friendly online application (https://prehaicnomogramforhcc.shinyapps.io/DynNomapp/) was developed.

**Fig. 3:**
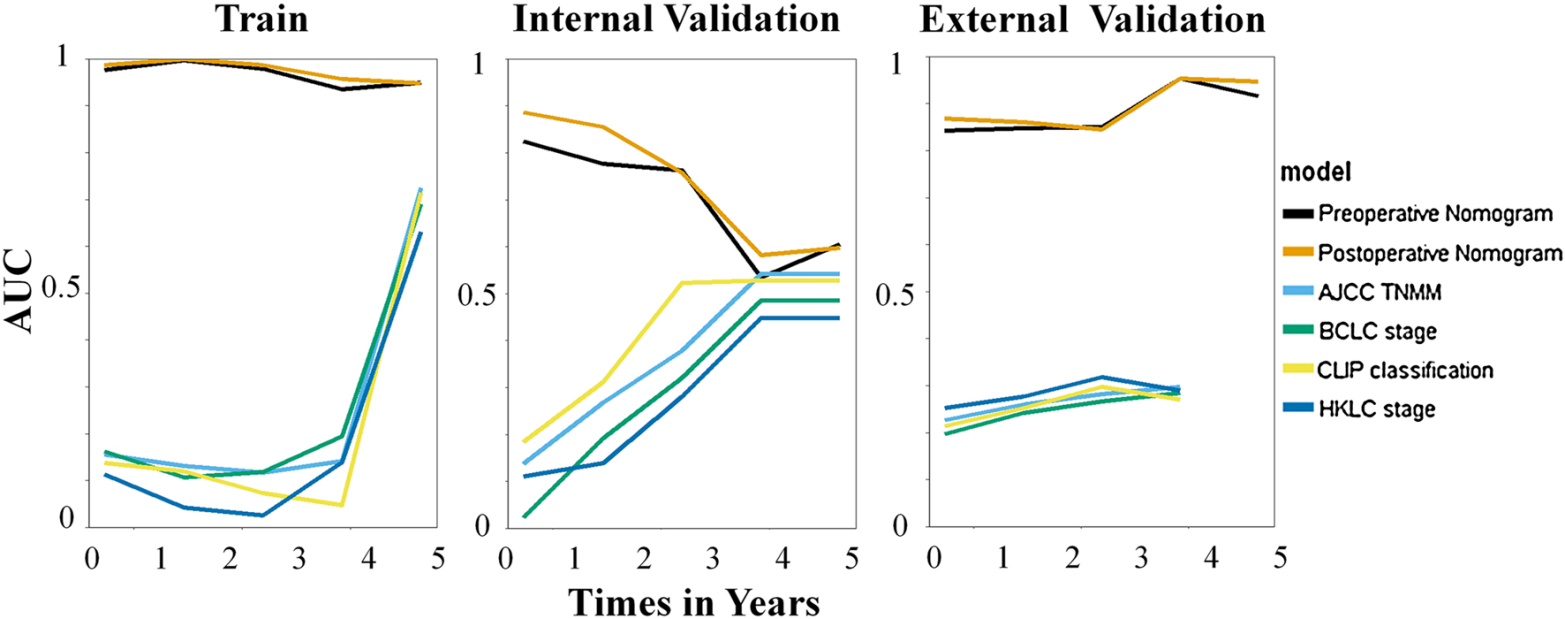
Discriminatory performance of all models and systems in thee cohorts. Graphs show time-dependent areas under the receiver operating characteristic (ROC) curve at various time points (top) for established models and staging systems. AJCC = American Joint Committee on Cancer, BCLC = Barcelona Clinic Liver Cancer, CLIP = Cancer of the Liver Italian Program, HKLC = Hong Kong Liver Cancer

The AUCs of the preoperative MDLRN for predicting the OS of HCC patients who underwent HAIC in the TC, IVC and EVC were 0.80, 0.71 and 0.74, respectively. In addition, the AUCs of the postoperative MDLRN for predicting OS in the TC, IVC and EVC were 0.84, 0.78 and 0.79, respectively. In this study, we found that the MDLRN improved the prognostic prediction of HCC patients who underwent HAIC compared with rival models and staging systems (AJCC [American Joint Committee on Cancer], BCLC [Barcelona Clinic Liver Cancer] stage, CLIP [Cancer of the Liver Italian Program] classification, HKLC [Hong Kong Liver Cancer] stage) in the three cohorts (Fig. 3).

### Visualization interpretability

**Fig. 4:**
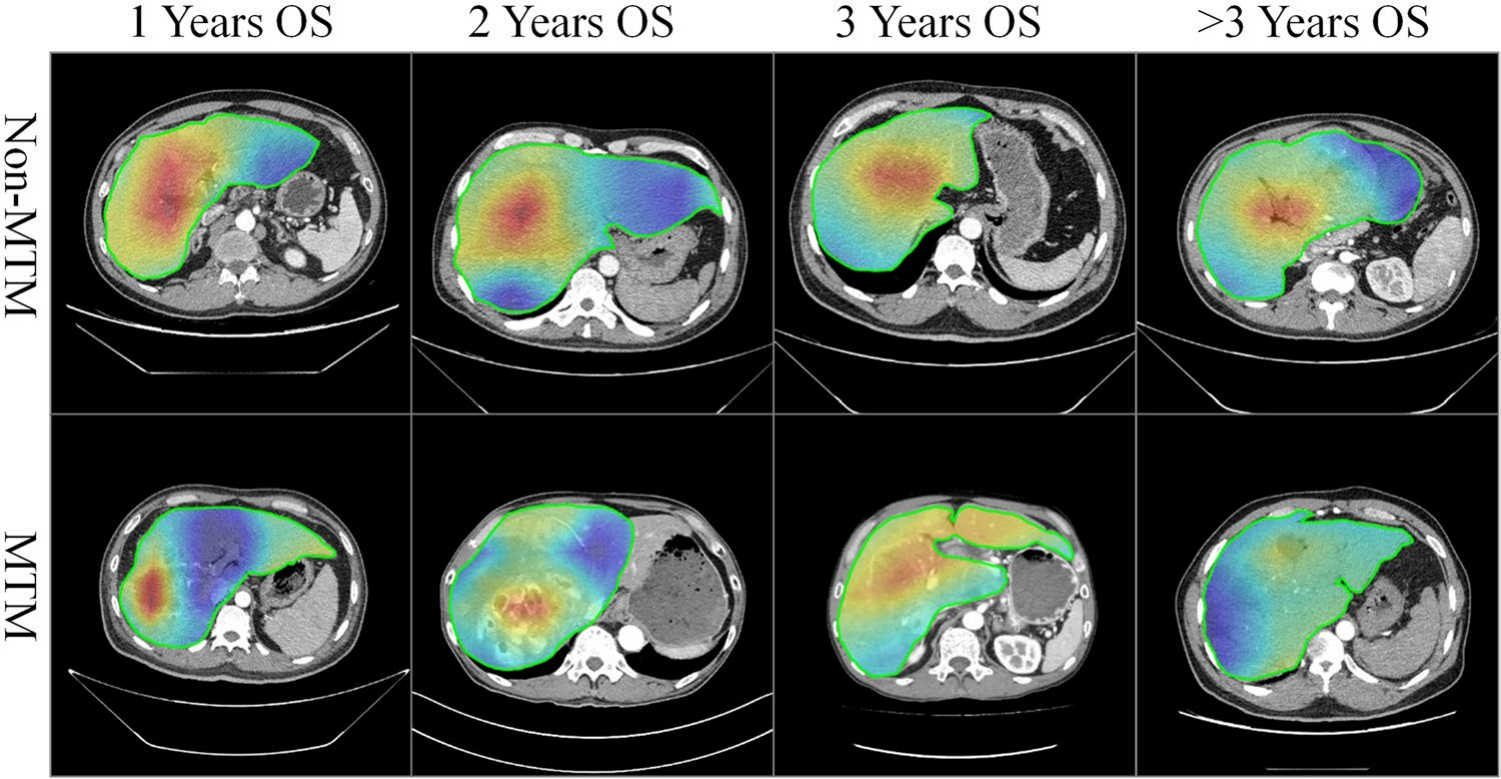
The learned feature maps of MobileNetV1 for 1-, 2-, 3-, > 3 years OS. The 1-year OS non-MTM image come from a patient with 56 years old, Portal Vein Tumor Thrombus (PVTT), Stable Disease (SD) and 6 HAIC session. The 1-year OS MTM image come from a patient with 40 years old, Portal Vein Tumor Thrombus (PVTT), Stable Disease (SD) and 2 HAIC session. The 2-years OS non-MTM image come from a patient with 60 years old, Portal Vein Tumor Thrombus (PVTT), Stable Disease (SD) and 5 HAIC session. The 2-years OS MTM image come from a patient with 63 years old, No Portal Vein Tumor Thrombus (PVTT), Stable Disease (SD) and 6 HAIC session. The 3-years OS non-MTM image come from a patient with 47 years old, Portal Vein Tumor Thrombus (PVTT), Partial Response (PR) and 3 HAIC session. The 3-years OS MTM image come from a patient with 58 years old, No Portal Vein Tumor Thrombus (PVTT), Progressive Disease (PD) and 2 HAIC session. The > 3-years OS non-MTM image come from a patient with 26 years old, Portal Vein Tumor Thrombus (PVTT), Partial Response (PR) and 6 HAIC session. The > 3-years OS MTM image come from a patient with 58 years old, No Portal Vein Tumor Thrombus (PVTT), Partial Response (PR) and 8 HAIC session

The learned feature maps of MobileNetV1 are shown in Fig. 4 and detailed patient information in sTable 7. To better explore the hidden patterns the network learned, heatmaps were divided into prediction groups for 1/2/3/ > 3-year death/survival. According to their imaging features, the examples were divided into MTM and non-MTM subtypes. Overall, the whole intensity of the feature map in the predicted non-MTM group was lower than that in the predicted MTM group, which seems to indicate the natural pathological characteristics of HCC. Moreover, heatmaps showed that the better survival group had a high intensity, which indicated that the MTM subtype was an important factor for prognostic analysis.

### Survival risk stratification

**Fig. 5:**
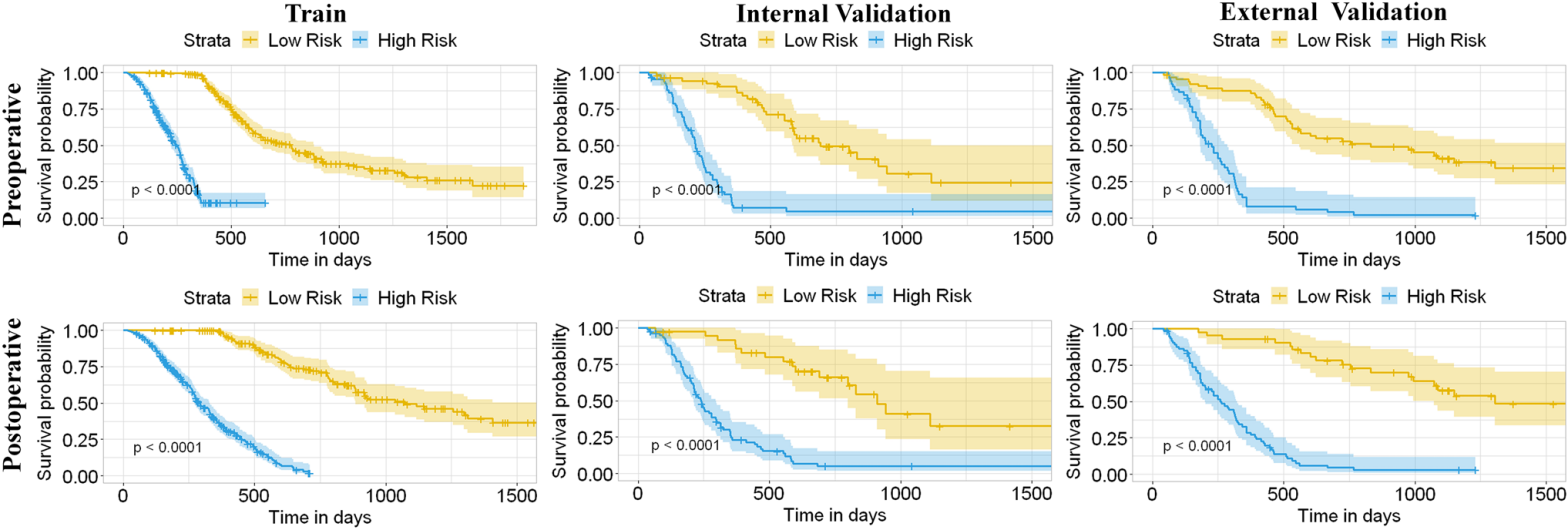
Comparing the survival among different risk level groups based on the two prognostic models. According to the risk scores from the pre-nomogram, the HCC patients were divided into high-, and low-risk groups **A**–**C**. Kaplan–Meier (KM) curves for the overall survival (OS) of HCC patients in these two risk level groups in **A** the training cohort, **B** the internal test cohort, **C** the first external test cohort. KM analysis of the risk scores for OS among the high-,and low-risk groups based on the post-nomogram (post-HAICN) in **D** the training cohort, **E** the internal test cohort, **F** the external test cohort

To facilitate the clinical application of the MDLRN, we divided the HCC patients who underwent HAIC into two risk groups, including a high-risk group and a low-risk group, according to MDLRN risk scores. We identified the HR cut-off values for the pre- and post-MDLRN (−1.40 and −0.26) in the TC and verified them in the ITC and ETC, respectively. This pragmatic visualization of the risk level could help decide the HAIC strategy for HCC patients. According to the cut-off risk scores for the pre-MDLRN, in the TC, the 1-, 3- and 5-year OS were 89.0%, 52.9% and 34.3% in the low-risk group, respectively, which were better than the corresponding rates in the high-risk groups (37.2%, 5.5% and 5.5%) (*p* < 0.001) (Fig. 5A). Similarly, the cumulative 1-, 3-, and 5-year OS rates among the high-risk and low-risk groups were also significantly different in the other two test cohorts (both, *p* < 0.001) (Fig. 5B, C). According to the cut-off risk scores for the post-MDLRN, in the TC, the 1-, 3- and 5-year OS were 91.2%, 55.4% and 25.1% in the low-risk group, respectively, which were better than the rates in the high-risk group (35.7%, 3.8% and 3.8%, respectively) (*p* < 0.001) (Fig. 5D). Similarly, the cumulative 1-, 3-, and 5-year OS rates among the high-risk and low-risk groups were also significantly different in the other two test cohorts (both, *p* < 0.001) (Fig. 5E, F). In brief, more deaths were more commonly found during the follow-up period in high-risk patients than in low-risk patients; a higher proportion of low-risk patients received potentially curative therapy (liver transplant, repeat liver resection, or ablation) than high-risk patients.

## Discussion

According to current guidelines, the standard treatment for advanced HCC is sorafenib. HAIC is now being applied, mostly in Asia. However, large randomized trials are still lacking. In the EACH and other previous studies, the value of systemic chemotherapy with the FOLFOX regimen for the treatment of advanced HCC was confirmed [20–22]. The continuous infusion of chemotherapeutic drugs by hepatic intra-arterial therapy contributed to the adequate local drug concentration for targeted tumours. Previous studies have reported an encouraging safety profile and antitumour activity when treating locally advanced HCC with FOLFOX–HAIC [23, 24]. However, high-level tumour heterogeneity at the histologic, genomic, and molecular levels results in a certain range of individualized differences in the prognosis of HCC patients who undergo HAIC. Therefore, we developed and validated an MDLRN integrating clinical variables, DLR score and histologic features to provide indicators for selecting HAIC and therapeutic strategies in large HCC.

The MDLRN was constructed by a multiple-task deep learning model, clinical factors and radiomics features. The multiple-task deep learning model was constructed by MobileNetV1 feature extraction and 5 classification tasks (MTM prediction, 1-, 2-, 3- and greater than 3-years OS. Multitask deep learning has taken the pathological features of the MTM subtype into consideration. Thus, the same extraction module was used in the MTM scoring task and OS scoring tasks, further demonstrating the relationship between MTM and OS. Moreover, the postoperative pathological features could be predicted by the preoperative model, which can improve the performance of the preoperative model.

In this study, we aimed to identify a certain histologic subtype of HCC, which was defined as the MTM subtype. Notably, the incidence of this histologic subtype increased with increasing tumour diameter in previous studies. In MTM related cohort with 159 patients who underwent SR, 74.2% (118/159) of patients were diagnosed pathologically with the MTM subtype. We developed a DLR-based model for predicting the MTM subtype that showed outstanding performance (AUC, 0.98 in the TC, 0.84 in the IVC and 0.72 in the EVC). In addition, our results showed that a high serum AFP level was an independent predictor of the MTM subtype [11], which was consistent with previous reports. However, no incremental increase in value was observed with the addition of the DLR model to predict the MTM subtype. We also showed that a low baseline DLR score for MTM status was associated with OS (HR, 0.85; *p* < 0.001) in patients with large HCC who underwent HAIC, indicating its potential clinical application.

This study developed and validated an MDLRN for predicting the OS of patients with large HCC receiving initial HAIC based on CECT data from 752 patients in OS cohort, and the model could accurately stratify patients with large HCC into two prognostic subgroups with significantly different OS. In this study, three attempts were made, as follows: first, we built the MDLRN comprising preoperative and postoperative clinical variables, DLR signatures and MTM subtype for the prediction of OS; second, the entire liver parenchyma was automatically segmented as an ROI using the ResU-Net algorithm for feature extraction; third, we provided an MDLRN-based system as a visualized web tool to recommend suitable patients with large HCCs for HAIC treatment, achieving a good predictive performance (AUC, 0.87 in IVC; AUC, 0.83 in the EVC).

The DLR analysis highlighted the potential important roles of tumour burden and distribution in the entire liver parenchyma as well as the tumour microenvironment (TME) in prognostic prediction. Exposure of the targeted tumours to chemotherapy drugs over multiple cycles is closely related to treatment response [25]. Previous studies have suggested that a larger tumour burden and more dispersed distribution both weaken the effect of chemotherapy [4]. Similarly, our DLR visualization results were consistent with the abovementioned hypothesis. The predicted death group had a higher intensity heatmap than the predicted survival group, suggesting the importance of tumour burden and distribution. Moreover, previous studies exploring the mechanisms of conventional chemotherapy resistance have revealed the involvement of TME components and seem to explain the relationship between the status of the TME and the response to chemotherapy [26, 27]. In our heatmap, a higher intensity distribution may be consistent with TME component assembly, including for the ECM, proteoglycans, immune cells and hypoxic environment. This hypothesis needs further experimental research on the underlying mechanism.

The MDLRN based on preoperative DLR scores and clinical parameters should be useful for patient stratification before HAIC, allowing clinicians to optimize treatment, such as switches to SR and LT. Once the patients undergoes HAIC, the post-MDLRN, which was built with postoperative clinical parameters, including number of sessions of HAIC, response to HAIC and predicted MTM subtype, has significantly higher predictive performance and can be used to design individualized surveillance and therapeutic strategies. Through patient stratification performed by the MDLRN, an intensive surveillance regimen, and even some aggressive or expensive preventive and adjuvant therapies, including preventive multitargeted tyrosine kinase inhibitors [TKIs] and programmed cell death protein (PD)-1 therapies, can be considered to prolong the OS of high-risk patients [28, 29]. On the other hand, low-risk patients may receive less intensive surveillance regimens and more prudent consideration of aggressive or expensive preventive therapies after HAIC to reduce the probability of negative effects and the high cost of these examinations and therapies.

There are some limitations to our study. First, selection bias is unavoidable in observational studies and may affect the real outcomes. Second, we did not perform manual delineation of the tumour area to extract features. Whether the predictive ability of the MDLRN model would significantly improve over that of a model based on the entire tumour ROI remains to be further tested in external cohorts. Third, as time progresses, the therapeutic techniques for HCC are constantly being updated and improved, such as the adjustment of HAIC chemotherapy drug regimens and the improvement of HAIC combined with molecular targeted drugs. This will have a certain degree of impact on outcome prediction and is inevitable Fourth, clinical information regarding complications during and after HAIC and TKI treatment were not analysed, warranting further investigation. Given these limitations, the MDLRN model requires further validation as an OS stratification tool for HAIC in patients with HCC before being applied in other study settings.

In conclusion, MTM is an important prognosis factor for HCC patients which was taken into consideration for building the multitask DLR method. The model could predict the prognosis of HCC patients who underwent HAIC and showed excellent performance in two test cohorts, demonstrating its robustness and effectiveness. Therefore, this tool may help physicians with therapeutic decision making and surveillance strategy selection in clinical practice.

### Supplementary Information

Below is the link to the electronic supplementary material.

Supplementary file1 (DOCX 132 KB)

## Data Availability

The in-house developed medical database of this study is publicly accessible at:http://www.yunedc.cn/#/login
