# Correction: A multitask deep learning radiomics model for predicting the macrotrabecular‑massive subtype and prognosis of hepatocellular carcinoma after hepatic arterial infusion chemotherapy

**DOI:** 10.1007/s11547-023-01754-y

**Published:** 2024-01-17

**Authors:** Xuelei He, Kai Li, Ran Wei, Mengxuan Zuo, Wang Yao, Zechen Zheng, Xiaowei He, Yan Fu, Chengzhi Li, Chao An, Wendao Liu

**Affiliations:** 1 Department of Interventional Therapy, Guangdong Provincial Hospital of Chinese, Medicine and Guangdong Provincial Academy of Chinese Medical Sciences, No. 111 Dade Road, Guangzhou, 510080 Guangdong People’s Republic of China; 2 School of Information Sciences and Technology, Northwest University, Xi’an, 710127 Shaanxi Province People’s Republic of China; 3 Department of Minimal Invasive Intervention, Sun Yat-sen University Cancer Center; State Key Laboratory of Oncology in South China, Collaborative Innovation Center for Cancer Medicine, 651, Dongfeng East Road, Guangzhou, 510060 People’s Republic of China; 4 Department of Ultrasound, The Third Affiliated Hospital of Sun Yat-sen University, Guangzhou, 510080 Province Guangdong People’s Republic of China; 5 Department of Interventional Radiology and Vascular Surgery, The Third Affiliated Hospital of Sun Yat-sen University, Guangzhou, 510080 Province Guangdong People’s Republic of China; 6 Department of Interventional Radiology and Vascular Surgery, The First Affiliated Hospital of Jinan University, Guangzhou, 510060 People’s Republic of China; 7 Department of Interventional Therapy, National Cancer Center/National Clinical, Research Center for Cancer/Cancer Hospital, Chinese Academy of Medical, Sciences and Peking Union Medical College, Beijing, 100021 People’s Republic of China

**Correction to: La radiologia medica (2023) 128:1508–1520** 10.1007/s11547-023-01719-1

In the original publication of the article, the affiliations of the fifth and nineth author were published incorrectly as below:

Wang Yao—Department of Interventional Radiology and Vascular Surgery, The First Affiliated Hospital of Jinan University, Guangzhou 510060, People's Republic of China

Chengzhi Li—Department of Ultrasound, The Third Affiliated Hospital of Sun Yat-sen University, Guangzhou 510080, Province Guangdong, People's Republic of China

The correct affiliation is as follows:

Wang Yao—Department of Interventional Radiology and Vascular Surgery, The Third Affiliated Hospital of Sun Yat-sen University, Guangzhou 510080, Province Guangdong, People's Republic of China

Chengzhi Li—Department of Interventional Radiology and Vascular Surgery, The First Affiliated Hospital of Jinan University, Guangzhou 510060, People's Republic of China

The original article has been corrected.

