# Supplementary material for: A multitask deep learning radiomics model for predicting the macrotrabecular-massive subtype and prognosis of hepatocellular carcinoma after hepatic arterial infusion chemotherapy
Source: Radiol Med. 2023 Oct 6;128(12):1508–20. doi: 10.1007/s11547-023-01719-1 (PMC10700409; doi:10.1007/s11547-023-01719-1)
Supplement: Supplementary file 1 — Supplementary file1 (DOCX 132 KB) [file 11547_2023_1719_MOESM1_ESM.docx]

**Supplementary Material for “A Multitask Deep Learning Radiomics Model for Predicting the Macrotrabecular-Massive Subtype and Prognosis of Hepatocellular Carcinoma after Hepatic Arterial Infusion Chemotherapy”**

**This supplementary material includes:**

1. **Supplementary Methods**

**E1.1** The reasons for conducting HAIC rather than TKIs

**E1.2** HAIC procedure

**E1.3** Criteria for protocol treatment discontinuation

**E1.4** CECT scan protocol

**E 1.5** Definitions of Variables

**E1.6** The process of automatic delineation

**E1.7** MDL Model Construction

**E1.8** Radiomic Model Construction of MTM

**E1.9** The procedure of DLR-Cli model

1. **Supplementary Tables**

**sTable 1** Multi-task MobilenetV1 Structure

**sTable 2** The clinicoradiologic characteristics of HCC in two cohorts.

**sTable3** Baseline characteristics of patients with MTM subtype

**sTable 4** Prognostic Performance of DL-based Models for prediction of MTM

**sTable 5** Multivariable Regression Analysis of Predictors of OS in the Primary Cohort

**sTable 6** The performance of MobilenetV1 in OS time Class

**sTable 7** The detailed information of patient for DL visualization

1. **Supplementary Figures**

**sFigure 1** Detailed structures of different convolution

1. **Supplementary Methods**

E1.1 The reasons for conducting HAIC rather than TKIs

1. Cost is too high to afford;

ii) Positive recommendations from medical experts.

**E1.2 HAIC procedure**

HAIC procedures have been described in a serial of previous studies[1-3]. All procedures were performed using digital subtraction angiography (Philips, type FD 20 1250 mA, Amsterdam, Netherlands). The artery sheath catheter was inserted into the femoral artery using the modified Seldinger technique. A 5-Fr Yashiro catheter (Terumo, Tokyo, Japan) was advanced into the celiac trunk and superior mesenteric artery to assess the feeding hepatic artery. A 2.7-Fr micro-catheter (Terumo, Tokyo, Japan) was inserted in the feeding artery. If the tumors simultaneously accept blood supply from the celiac trunk and superior mesenteric artery, the microcatheter will be placed into the largest tumor feeding arteries. The peripheral end of the micro-catheter will be locked with a heparin lock (10 ml, 10,000 units, 1: 1,000 dilution) to prevent clotting of the catheter. The peripheral part of the catheter exposed outside the body will be covered with medical sterile gauze and fastened on the skin of the thigh using medical rubberized fabric and a bandage.

**E1.3 Criteria for protocol treatment discontinuation**

1. Tumor progression

The progression disease (PD) were assessed by dynamic CT or MRI based on modified Response Evaluation Criteria in Solid Tumor (mRECIST).

1. Intolerable adverse event

i) Patient cannot resume HAIC after 30 days of interruption due to an adverse event;

ii) An adverse event that meets the criteria for HAIC dose reduction occurs after the dose was already reduced to the lowest level;

iii) Life-threatening adverse event;

C)The need for another anticancer treatment due to downstaging at the physician’ s discretion;

D) HAIC becomes technically infeasible;

E) Patient requests to discontinue the study；

F) Death.

E1.4 CT scanning protocol

All CT exams were performed with a standard protocol, using a 64-row detector scanner (Somatom Sensation CT, Siemens Medical Systems). The scanning parameters were 1.2 × 24 collimation, 120 kV (peak), 140–240 mAs (using automated dose modulation), 5.0 mm slice thickness with a reconstruction interval of 2.0 mm. All patients received intravenous non-ionic CA (Ultravist 370, Bayer HealthCare Pharmaceuticals; 370 mg of iodine/1 ml), at a volume of 1.4 ml/kg of body weight, by a bolus at 3 ml/s, using a mechanical power injector (Medrad Stellant CT Injection System), followed by a 40 ml saline flush through a 20-G catheter inserted into an antecubital vein. The single-level monitoring low-dose scanning (20 mAs) was initiated 5 s after CA injection, and arterial phase scanning was started automatically 15 s after the trigger threshold (increase of 120 Hounsfield units (HU)) had been reached at the level of suprarenal abdominal aorta. Portal venous (extended to the chest and lower abdomen) and equilibrium-phase acquisitions were obtained at 70 s and 180 s, respectively.

**E 1.5 Definitions of Variables**

In this study, 28 clinical variables are collected as follows: (1) demographic and history variables (ECOG, age, gender, comorbidities (i.e., hypertension, diabetes, heart disease, renal disease a esophageal gastric varices, etc.), etiology, CTP class, ALBI grade, ascites); (2) tumor features (maximal tumor diameter, number of tumor, tumor burden, PVTT, metastasis); (3) laboratory findings (α-fetoprotein [AFP], serum albumin; [ALB], total bilirubin [TB], platelet counts, prothrombin time (PT), international normalized ratio [INR], aspartate aminotransferase [AST] and alanine aminotransferase [ALT]), C reactive protein (CRP), creatinine, neutrophils, lymphocyte. Albumin- bilirubin (ALBI) grades were used to replace CTP grade for their objectiveness. ALBI score was calculated before treatment using the appropriate clinical parameters and ALBI grade was defined as follows: (log 10 bilirubin [BI] [μ mol /L] × 0.66) + (albumin [AL] [g/L] ×-0.085), (grade 1, 2, and 3 = ≤ -2.60, > -2.60 to -1.39, and > -1.39, respectively). For more detailed evaluations of patients with the middle grade of ALBI (grade 2), we used modified ALBI (mALBI) grading consisting of 4 levels, which included subgrading for the middle grade of 2 (2a and 2b) based on an ALBI score of –2.27 as the cut-off, which was previously reported as the value for indocyanine green retention after 15 min (ICG-R15) of 30%[4] ; (4) treatment parameters (HAIC sessions, sequential local therapy and the response of first HAIC). The responses was assessed by dynamic contrast enhanced CT or magnetic resonance imaging (MRI) based on modified Response Evaluation Criteria in Solid Tumor (mRECIST), including complete response (CR), partial response (PR), stable disease (SD), and progression disease (PD), which was performed every 4–6 weeks after initial IAT and evaluated independently by two radiologists (reader 1, L.Z.L., and reader 2, J. Z., with 10 years of experience) who were blinded to HAIC procedures at the time of data collection.

**E1.6 The process of automatic delineation**

Among a lot of deep learning-based segmentation methods, ResU-Net [5] was employed to segment the liver parenchyma and tumor areas because of its high accuracy and strong generalization characteristics. ResU-Net could configure itself and choose the most suitable model parameters based on different datasets. As we had the same data modality and segmentation task, ResU-Net pretrained on the Liver and Liver Tumor Segmentation challenge (LiTS) dataset [6] was selected in this study. Then, all data of this study were segmented by the ResU-net model.

**E1.7 MDL Model Construction**

MDL model is mainly constructed by a 3D MoblieNetV1 structure.

In 3D MobileNetV1, a single filter is applied to each input channel of the deep convolutional network. Then, pointwise convolution applies a 1*1*1 convolution network to merge the output of deep convolution. This standard convolution method can both filter and merge the input into a new set of output in one step. In this, the depthwise separable convolution divides it into two layers, one for filtering and the other for merging. The network structure of MobileNet is as follows. It consists of 28 layers (excluding AvgPool and FC layers, and separates deep convolution and point-by-point convolution). In addition to the standard convolution kernel used in the first layer, the remaining convolution layers are depthwise separable Convolution. (**sTable 1**)

**Batch normalization layer**

Batch normalization (BN) [7] is a non-parametric operation, which is used to accelerating the training process and deal with the over-fit problem. The BN is a practical method to regularizes the model and to enable higher learning rates. The formulation can be described as:

$\hat{x}=\frac{x-E\left[ x \right]}{\sqrt{Var\left[ x \right]+\epsilon}}$ *(1)*

Where $E\left[ x \right]$ is the expectation over training mini-batches, and $Var\left[ x \right]=\frac{m}{m-1}\dot{E_{B}}\left[ \sigma_{B}^{2} \right]$ is the unbiased variance estimate over training mini-batches of size $m$ and sample variances $\sigma_{B}^{2}$.

**Non-linear activation**

Non-linear activation is a non-linear function which can transform the linear system to a non-linear one. The operation is usually a map function to enhance fitting ability of model. ReLU6 function [8] are utilized in MobilenetV1, and the operations is element-wise.

The ReLU6 function is expressed as

$relu6(x)=\min(\max(x,0),6)\in[0,6]$ (2)

and this operation is utilized in each Convolution Module.

**Pooling**

The pooling operation is a method to reduce the parameters and redundant information. Because of the static property of image, local features can be described by statistics of local region. Thus, the operation paid more attention to the whole image domain and ignored the local information, which lead to the invariance in translation, rotation and scale. In this study, we use 3D avg pooling with strides 8*8*1 to construct the Convolution Module.

**Convolution Module**

In this study, traditional 3D convolution module is utilized, which is constructed in order of 3D Convolution operation, BN, non-linear activation and pooling.

**Depthwise Separable Convolution**

Depthwise separable convolution [9] is actually a factorized convolution operation. It can be decomposed into two smaller operations: depthwise convolution and pointwise convolution.

For a standard convolution (**Figure S1A**), an input feature map of 12 * 12 * 3 is input, and an output feature map of 8 * 8 * 1 is obtained by 5 * 5 * 3 convolution kernel. If we have 256 feature maps at this time, we will get an 8 * 8 * 256 output feature map, as shown in Figure 1.

For deep convolution (**Figure S1B**), all the feature map channels are decomposed, each feature map is a single channel mode, and each single channel feature map is convoluted. In this way, the generated feature map with the same number of channels as the original feature map will be obtained. Assuming that the input feature map of 12 * 12 * 3 is input, after 5 * 5 * 1 * 3 deep convolution, the output feature map of 8 * 8 * 3 is obtained. The input and output dimensions are constant 3, so there will be a problem that the number of channels is too small, the dimension of the feature map is too small, and the information cannot be effectively obtained.

Pointwise convolution (**Figure S1C)** is 1 * 1 convolution, and its main function is to increase the dimension and reduce the dimension of the feature map. In the process of deep convolution, we get the output feature map of 8 * 8 * 3. We use 256 1 * 1 * 3 convolution kernels to convolve the input feature map. The output feature map is 8 * 8 * 256 just like the standard convolution operation. The following figure:

The process of deepwise separable convolution is Figure S1D.

**Fully connected (FC) layers**

The function of the fully connected layer is to classify and map the learned "patrial feature representation" to the sample label space. The fully connected layer can be described as matrix multiplication plus a bias term, the formula is as follows

$FC\left( x \right)=Wx+b$  *(4)*

The fully connected layer is usually used at the top of the network to provide a global receptive field for the network to overcome the shortcomings that the convolutional layer has only limited receptive fields. Through the fully connected layer, the feature maps can be merged and compressed at the same time, and converted into the probability output of each category. In this study, the fully connected layer is used during deep learning model training and testing.

**Network Training**

The loss function of the network is shown below:

$\text{L}=\frac{1}{\text{N}}\sum_{i} L_{i}=-\frac{1}{\text{N}}\sum_{i} \sum_{c=1}^{M} y_{ic}\log(p_{ic})$ *(5)*

where $M$ is Number of categories and $y_{ic}$ is the symbol function (0 or 1), if the true category of sample i is equal to c take 1, otherwise take 0. *p_ic_* is the prediction probability of observation sample i belonging to category c.

We used Adam [10] as the optimizer with a batchsize of 16. We used the learning rate of 0.0001 and trained for 200 epochs. Data augmentation including random rotation, flip, and crop was applied to reduce the risk of overfitting. We then adopted 5-fold cross validation to find the best hyper-parameter setting.

**E1.8 Radiomic Model Construction of MTM**

Radiomics model was constructed by pre-defined radiomic features, extracted based on the commonly used pyradiomics including shape-based, first order statistics, and texture features (Gray Level Co-occurrence Matrix (GLCM) Features, Gray Level Size Zone Matrix (GLSZM) Features, Gray Level Run Length Matrix (GLRLM) Features, Neighbouring Gray Tone Difference Matrix (NGTDM) Features, Gray Level Dependence Matrix (GLDM) Features. And radiomic feature selection was conducted only in the training cohort. First of all, the variance threshold method was used and the features with eigenvalues of variance less than 0.02 were removed. Second, P-value was calculated by analysis of variance (ANOVA) to select the top 50 features with the highest F-value between different labeled groups. Then, correlation comparation was employed to eliminate the correlation between features. More specifically, compare all the selected features in pairs and if the Pearson correlation coefficient of the two features is higher than 0.95, the feature with lower F-value will be removed. Finally, we performed the XGBoost to establish the classification model of MTM, and select the most important features which are significantly associated with treatment response. Five-fold cross-validation was repeated 10 times to avoid bias resulting from the initial data split.

For 2510 pre-defined radiomic features in Macrotrabecular-massive (MTM) subtype of hepatocellular carcinoma (HCC), 709 features were selected after variance filter, 50 features were selected after SelectKBest, 33 features were selected after correlation comparation and only 22 features left after XGBoost.

For pre-defined radiomic signature building of MTM, the extreme gradient boosting (XGBoost) classifier was used for 5-fold cross validation in the training cohort. The input features were determined using the above feature selection. The performance of the Radiomics method is shown in **sTable 4**.

**E1.9 The procedure of DLR-Cli model**

The DLR-Cli model was based on Clincial, Multi-tasks Deep Learning and Radiomics models. 6 Clincial factors was selected from 22 features by univariate analysis and multivariate analysis, which constructed the clinical model by logistic regression. The radiomics scores can be expressed as:

Clinical score= 1.0859+0.0239* Tumor size -0.7938*PVTT+0.7915* Metastasis - 0.1950*Local Treatment - 1.6552*HAIC_TACE_SR - 1.0188* HAIC_MWA + 1.8794*OR

In addition, we also extracted the deep learning features for hidden information and radiomic scores from radiomics features as important supplementing. The Detailed construction and training process of our proposed model are described as above **E1.6-E1.7**. And the three model scores were combined with following formulation:

DLR-Cli = 0.2*DL Score + 0.6*Radiomics Score + 0.2*Clinical Score

**Reference**

1. Ueshima K, Komemushi A, Aramaki T, Iwamoto H, Obi S, Sato Y, Tanaka T, Matsueda K, Moriguchi M, Saito H, Sone M, Yamagami T, Inaba Y, Kudo M, Arai Y. Clinical Practice Guidelines for Hepatic Arterial Infusion Chemotherapy with a Port System Proposed by the Japanese Society of Interventional Radiology and Japanese Society of Implantable Port Assisted Treatment. Liver Cancer. 2022 May 5;11(5):407-425. doi: 10.1159/000524893.

2. Yamasaki T, Saeki I, Yamauchi Y, Matsumoto T, Suehiro Y, Kawaoka T, Uchikawa S, Hiramatsu A, Aikata H, Kobayashi K, Kondo T, Ogasawara S, Chiba T, Takami T, Chayama K, Kato N, Sakaida I. Management of Systemic Therapies and Hepatic Arterial Infusion Chemotherapy in Patients with Advanced Hepatocellular Carcinoma Based on Sarcopenia Assessment. Liver Cancer. 2022 Feb 22;11(4):329-340. doi: 10.1159/000522389.

3. Wang T, Dong J, Zhang Y, Ren Z, Liu Y, Yang X, Sun D, Wang Y. Efficacy and safety of hepatic artery infusion chemotherapy with mFOLFOX in primary liver cancer patients with hyperbilirubinemia and ineffective drainage: a retrospective cohort study. Ann Transl Med. 2022 Apr;10(7):411. doi: 10.21037/atm-22-978.

4.Hiraoka A, Kumada T, Tsuji K, Takaguchi K, Itobayashi E, Kariyama K, et al. Validation of Modified ALBI Grade for More Detailed Assessment of Hepatic Function in Hepatocellular Carcinoma Patients: A Multicenter

Analysis. Liver Cancer. 2019 Mar;8(2):121–129.

5. Diakogiannis F I, Waldner F, Caccetta P, et al. ResUNet-a: A deep learning framework for semantic segmentation of remotely sensed data. ISPRS Journal of Photogrammetry and Remote Sensing, 2020, 162: 94-114.

6. Bilic P, Christ P, Li H B, et al. The liver tumor segmentation benchmark (lits). Medical Image Analysis, 2023, 84: 102680.

7. Ioffe S, Szegedy C. Batch normalization: Accelerating deep network training by reducing internal covariate shift. International conference on machine learning. pmlr, 2015: 448-456.

8. Krizhevsky A, Hinton G. Convolutional deep belief networks on cifar-10. Unpublished manuscript, 2010, 40(7): 1-9.

9. Chollet F. Xception: Deep learning with depthwise separable convolutions. Proceedings of the IEEE conference on computer vision and pattern recognition. 2017: 1251-1258.

10. Kingma D P, Ba J. Adam: A method for stochastic optimization. International Conference on Learning Representations. 2015.

1. **Supplementary Tables**

sTable 1 Multi-task MobilenetV1 Structure

| **Type/Stride** | | **Filter Shape** | | | | |
| --- | --- | --- | --- | --- | --- | --- |
| Conv 3d / s2 | | 3 × 3 × 3 × 32 | | | | |
| Conv 3d dw / s1 | | 3 × 3 × 3 × 32 | | | | |
| Conv 3d / s1 | | 1 × 1 × 1 × 32 × 64 | | | | |
| Conv 3d dw / s2 | | 3 × 3 × 3 × 64 | | | | |
| Conv 3d / s1 | | 1 × 1 × 1 × 64 × 128 | | | | |
| Conv 3d dw / s1 | | 3×3×3×128 | | | | |
| Conv 3d / s1 | | 1 × 1 × 1 × 128 × 128 | | | | |
| Conv 3d dw / s2 | | 3 × 3 × 3 × 128 | | | | |
| Conv 3d / s1 | | 1 × 1 × 1 × 128 × 256 | | | | |
| Conv 3d dw / s1 | | 3 × 3 × 3 × 256 | | | | |
| Conv 3d / s1 | | 1 × 1 × 1 × 256 × 256 | | | | |
| Conv 3d dw / s2 | | 3 × 3 × 3 × 256 | | | | |
| Conv 3d / s1 | | 1 × 1 × 1 × 256 × 512 | | | | |
| 5× | Conv 3d dw / s1 | 3 × 3 × 3 × 512 | | | | |
|  | Conv 3d / s1 | 1 × 1 × 1 × 512 × 512 | | | | |
| Conv 3d dw / s2 | | 3 × 3 × 3 × 512 | | | | |
| Conv 3d / s1 | | 1 × 1 × 1 × 512 × 1024 | | | | |
| Conv 3d dw / s2 | | 3 × 3 × 3 × 1024 | | | | |
| Conv 3d / s1 | | 1 × 1 × 1 × 1024 × 1024 | | | | |
| Avg Pool / s1 | | 8 × 8 × 1 | | | | |
| FC / s1 | | 1024 × 1024 × 2 | 1024 × 1024 × 2 | 1024 × 1024 × 2 | 1024 × 1024 × 2 | 1024 × 1024 × 2 |
| Softmax / s1 | | Classifier | | | | |

Note. Conv 3d: 3d convolution ; s2: convolution stride is 2; s1: convolution stride is 1; Avg Pool: Average pooling ;FC: Full connection; dw: Depthwise Separable Convolution

| **sTable 2.** The clinicoradiologic characteristics of HCC in two cohorts. | | |
| --- | --- | --- |
| **Variables** | **SR, n = 159** | **HAIC, n = 752** |
| **Age (years)** |  |  |
| ≤ 65 | 147(92.45%) | 662(87.36%) |
| > 65 | 12(7.55%) | 90(12.64%) |
| **Sex** |  |  |
| Female | 24(15.09%) | 80(10.64%) |
| Male | 135(84.91%) | 672(89.36%) |
| **HBV** |  |  |
| Absence | 7(4.40%) | 64(8.51%) |
| Presence | 152(95.60%) | 688(91.49%) |
| **HCC number** |  |  |
| 1-3 | 87(54.72%) | 393(52.26%) |
| > 3 | 72(45.28%) | 359(47.74%) |
| **HCC diameter, cm** |  |  |
| ≤ 7 | 19(11.95%) | 92(12.23%) |
| > 7 | 140(88.05%) | 660(87.77%) |
| **ALBI grade** |  |  |
| 1 | 6(3.77%) | 166(22.07%) |
| 2-3 | 153(96.22%) | 586(77.93%) |
| **AFP, ng/ml** |  |  |
| ≤ 400 | 61(38.36%) | 270(35.90%) |
| > 400 | 98(61.64%) | 482(64.10%) |
| **BCLC stage** |  |  |
| A＆B | 59(37.11%) | 165(21.94%) |
| C | 100(62.89%) | 587(78.06%) |
| **MTM** |  |  |
| Absence | 41(25.79%) | NAN |
| Presence | 118(74.21%) | NAN |
| Note. Data are number of patients; data in parentheses are percentage of patients unless otherwise indicated. The data in two groups were compared by using the Chi square test.  Abbreviation: HAIC: HBV, viral hepatitis type B; AFP, α-fetoprotein; | | |

sTable 3 Baseline characteristics of patients with Macrotrabecular-Massive subtype

| **Variables** | **Training cohort**  **(n = 85)** | **Internal validation cohort**  **(n = 36)** | **External validation cohort**  **(n = 38)** | ***P* value** |
| --- | --- | --- | --- | --- |
| ***Demographics*** |  |  |  |  |
| Age (y), mean ± SD | 48±11.9 | 51±12.4 | 51±11.6 | 0.154 |
| Gender |  |  |  | 0.514 |
| Female | 14(16.47%) | 3(8.33%) | 7(18.42%) |  |
| Male | 71(83.53%) | 33(91.67%) | 31(81.58%) |  |
| BMI, mean ± SD | 21.23±2.36 | 21.65±2.53 | 21.55±2.86 | 0.672 |
| PS |  |  |  | 0.498 |
| ECOG 0 | 81(95.29%) | 34(94.44%) | 35(92.11%) |  |
| ECOG 1 | 4(4.71%) | 2(5.56%) | 3(7.89%) |  |
| Comorbidities |  |  |  | 0.497 |
| Absence | 76(89.41%) | 31(86.11%) | 32(84.21%) |  |
| Presence | 9(10.59%) | 5(13.89%) | 6(18.79%) |  |
| HBV |  |  |  | 0.232 |
| Absence | 2(2.35%) | 2(5.56%) | 3(7.89%) |  |
| Presence | 83(97.65%) | 34(94.44%) | 35(92.11%) |  |
| Ascites |  |  |  | 0.429 |
| Absence | 79 (92.94%) | 34(94.44%) | 34(89.47%) |  |
| Presence | 6(7.06%) | 2(5.56%) | 4(10.53%) |  |
| ALBI score, mean ± SD | -2.70±0.36 | -2.64±0.35 | -2.87±0.39 | 0.025 |
| Metastasis |  |  |  | 0.638 |
| Absence | 60(70.56%) | 21(58.33%) | 27(71.05%) |  |
| Presence | 25(29.44%) | 15(41.67%) | 11(28.95%) |  |
| LN |  |  |  |  |
| Lung | 5(5.88%) | 4 (11.11%) | 4(10.53%) |  |
| Adrenal | 0(0.00%) | 1(2.78%) | 0(0.00%) |  |
| Bone | 1(1.18%) | 0(0.00%) | 0(0.00%) |  |
| ***Image characteristics*** |  |  |  |  |
| Tumor size (cm)‎, mean±SD | 11.39±3.72 | 11.71±3.95 | 10.70±3.22 | 0.322 |
| No. of tumors |  |  |  | 0.173 |
| Single | 42(49.41%) | 14(38.89%) | 22(57.89%) |  |
| Multiple | 43(50.59%) | 22(61.11%) | 16(42.11%) |  |
| AFP (ng/ml)‎ |  |  |  | 0.948 |
| <400 | 35(41.18%) | 11(30.56%) | 15(39.47%) |  |
| ≥400 | 50(58.82%) | 25(69.44%) | 23(60.53%) |  |
| Median AST ‎(U/L)‎ | 61.5 | 70.2 | 51.5 | 0.178 |
| Median ALT ‎(U/L)‎ | 43.5 | 45.55 | 36.0 | 0.817 |
| Median TBIL (μmol/L)‎ | 14.3 | 17.75 | 11.55 | 0.264 |
| ALB (g/L), ‎mean±SD | 40.71±4.12 | 40.63±3.62 | 42.51±3.65 | 0.022 |
| INR, mean±SD | 1.07±0.10 | 1.08±0.09 | 1.04±0.08 | 0.144 |
| PT (s), mean±SD | 12.24±1.08 | 12.54±1.40 | 11.93±0.86 | 0.119 |
| Median PLT (×10^9^) | 232.0 | 220.5 | 272.5 | 0.820 |
| Cre (U/L), mean±SD | 69.01±18.02 | 70.50±17.05 | 78.79±76.27 | 0.583 |
| Median CRP (U/L) | 13.23 | 11.685 | 6.51 | 0.396 |
| Neu (μmol/L), mean±SD | 4.44±2.14 | 4.53±1.97 | 4.92±2.06 | 0.248 |
| Ly (μmol/L), mean±SD | 1.45±0.44 | 1.46±0.69 | 1.75±0.58 | 0.002 |
| ***Treatment and follow-up*** |  |  |  |  |
| Rounds of HAIC |  |  |  | 0.079 |
| 1 | 6(7.06%) | 5(13.89%) | 3(7.89%) |  |
| 2 | 20(23.53%) | 6(16.67%) | 13(34.21%) |  |
| 3 | 16(18.82%) | 6(16.67%) | 3(7.89%) |  |
| >3 | 43(50.59%) | 19(52.78%) | 19(50.00%) |  |
| Sequential treatment |  |  |  | <0.001 |
| None | 29(34.12%) | 19(52.78%) | 6(15.79%) |  |
| Surgery | 20(23.53%) | 13(36.11%) | 29(76.32%) |  |
| Ablation | 8(9.41%) | 5(13.88%) | 4(10.53%) |  |
| SBRT | 7(8.24%) | 0(0.00%) | 2(5.26%) |  |
| PD-1 | 18(21.17%) | 8(2.22%) | 5(13.16%) |  |
| TKI | 26(30.59%) | 13(36.11%) | 13(34.21%) |  |

Note. Data are number of patients; data in parentheses are percentage of patients unless otherwise indicated. The data in two groups were compared by using the Chi square test. Non-normally distributed data is represented by median and quartile. P value < 0.05 suggest statistically significant differences between three cohorts.

Abbreviation: HAIC: hepatic arterial infusion chemotherapy; FOLFOX, oxaliplatin plus fluorouracil and leucovorin; OR: objective responds; SD, standard deviation; BMI, body mass index; PS, performance status; ECOG, Eastern Cooperative Oncology Group; HBV, viral hepatitis type B; AFP, α-fetoprotein; ALBI, albumin-bilirubin; ALB, albumin; ALT, alanine aminotransferase; AST, aspartate aminotransferase; PT, prothrombin time; INR, international normalized ratio; TBIL, total bilirubin; PLT, platelet, SBRT, stereotactic body radiation therapy; TKI, tyrosine kinase inhibitor.

| **sTable 4. Prognostic Performance of DL-based Models for prediction of MTM** | | | | | | | |
| --- | --- | --- | --- | --- | --- | --- | --- |
| **Models** | **Cohorts** | **AUC** | **ACC** | **SENS** | **SPEC** | **PPV** | **NPV** |
| Clinical | Training | 0.6705  [0.6581, 0.7357] | 0.6705 [0.6366, 0.7037] | 0.6458 [0.6073, 0.6832] | 0.7496 [0.6893, 0.8085] | 0.8938 [0.8674, 0.9176] | 0.3950 [0.3626, 0.4292] |
|  | Internal  Test | 0.7344  [0.6981, 0.7716] | 0.7503 [0.7187, 0.7798] | 0.76938 [0.7341, 0.8033] | 0.6995 [0.6389, 0.7617] | 0.8700 [0.8455, 0.8930] | 0.5374 [0.4930, 0.5842] |
|  | External  Test | 0.6325 [0.5944, 0.6694] | 0.6318 [0.5976, 0.6657] | 0.6292 [0.5873, 0.6690] | 0.6359 [0.5778, 0.7024] | 0.8095 [0.7804, 0.8404] | 0.4115 [0.3742, 0.4462] |
| Radiomics | Training | 0.9742  [0.9614，0.9853] | 0.9765  [0.9647，0.9882] | 0.9873  [0.9768, 0.9967] | 0.9612  [0.9394, 0.9823] | 0.9749  [0.9597, 0.9883] | 0.9803  [0.9641, 0.9947] |
|  | Internal  Test | 0.8696  [0.8445, 0.8921] | 0.8749  [0.8518, 0.8969] | 0.9340  [0.9076, 0.9575] | 0.8052  [0.7598, 0.8450] | 0.8500  [0.8235, 0.8756] | 0.9117  [0.8822, 0.9407] |
|  | External  Test | 0.7356  [0.7007, 0.7701] | 0.8160  [0.7918, 0.8408] | 0.9259  [0.9042, 0.94645] | 0.5454  [0.4775, 0.6124] | 0.8333  [0.8136, 0.8542] | 0.7500  [0.6884, 0.8125] |
| DLR | Training | **0.9822**  **[0.9710, 0.9913]** | **0.9842**  **[0.9750, 0.9920]** | **0.9924**  **[0.9832, 1.0]** | **0.9722**  **[0.9531, 0.9876]** | 0.9813  [0.9687, 0.9916] | **0.9887**  **[0.9754, 0.9975]** |
|  | Internal  Test | 0.8392  [0.8122, 0.8668] | 0.8640  [0.8398, 0.8879] | **0.9487**  **[0.9265, 0.9673]** | 0.7297  [0.6770, 0.7778] | 0.847328  [0.82336182, 0.87121212] | **0.9000**  **[0.8630, 0.9335]** |
|  | External  Test | 0.7273  [0.6955, 0.7595] | 0.8421  [0.8228, 0.8609] | **1**  **[1, 1]** | 0.4545  [0.3875, 0.5190] | 0.8181  [0.8013, 0.8372] | **1**  **[1, 1]** |
| Clinical-DLR | Training | 0.9676  [0.9491, 0.9830] | 0.9770  [0.9650, 0.9870] | 0.98464 [0.9751, 0.9935] | 0.9500 [0.9149, 0.9787] | **0.9846 [0.9741, 0.9934]** | 0.9503 [0.9170, 0.9782] |
|  | Internal  Test | **0.9120**  **[0.8879, 0.9329]** | **0.91678 [0.8969, 0.9369]** | 0.9233 [0.9003, 0.9446] | **0.9002 [0.8592, 0.9386]** | **0.9603 [0.9446, 0.9752]** | 0.8184 [0.7743, 0.8611] |
|  | External  Test | **0.7733**  **[0.7405, 0.8080]** | **0.8687 [0.8498, 0.8889]** | **1 [1, 1]** | **0.5440 [0.4775, 0.6125]** | **0.8438 [0.8256, 0.8627]** | **1 [1, 1]** |
| **Note**. Numbers in parentheses are the 95% confidence interval.  **Abbreviation:** DL, deep learning; AUC, areas under receiver operating characteristic curve; ACC, accuracy; SENS, sensitivity; SPEC, specificity; PPV,  positive predictive value; NPV, negative predictive value. | | | | | | | |

| **sTable 5** Multivariable Regression Analysis of Predictors of OS in the Primary Cohort | | | | | | | | |
| --- | --- | --- | --- | --- | --- | --- | --- | --- |
| **Preoperative Model** | | | | **Postoperative Model** | | | | |
| **Variables** | **β** | **Odds Ratio (95% CI)** | **P value** | | **Variables** | **β** | **Odds Ratio (95% CI)** | **P value** |
| PVTT | 1.420 | [1.112,1.815] | 0.012 | | PVTT | 1.376 | [1.073,1.764] | 0.012 |
| DL-score | 0.112 | [0.087, 0.144] | <0.001 | | DL-score | 0.103 | [0.079, 0.135] | <0.001 |
| HAIC sessions | NA | NA | NA | | HAIC sessions | 0.737 | [0.662, 0.820] | <0.001 |
| OR | NA | NA | NA | | OR | 0.542 | [0.395,0.745] | <0.001 |
| MTM | NA | NA | NA | | MTM | 2.435 | [1.733,3.420] | <0.001 |

PVTT, Portal Vein Tumor Thrombus; OR, objective responds; MTM, macrotrabecular-massive

sTable 6 The performance of MobilenetV1 in OS time Class

| **Cohorts** | Acc | AUC | MCC | F1 | Kappa |
| --- | --- | --- | --- | --- | --- |
| Training | 0.9798  [0.9689,0.9889] | 0.9826  [0.9708,0.9919] | 0.9531  [0.9296,0.9741] | 0.9846  [0.9753,0.9924] | 0.9532  [0.9285,0.9741] |
| Internal Test | 0.8552  [0.8298,0.8799] | 0.8720  [0.8467,0.8979] | 0.6818  [0.6352,0.7264] | 0.8830  [0.8621,0.9040] | 0.6660  [0.6160,0.7147] |
| External Test | 0.8200  [0.7927,0.8468] | 0.8358  [0.8063,0.8651] | 0.5945  [0.5460,0.6420] | 0.8612  [0.8387,0.8837] | 0.5620  [0.5121,0.6135] |

sTable 7 The detailed information of patient for DL visualization

| **OS Time** | **MTM** | **Age** | **PVTT** | **OR** | **HAIC session** |
| --- | --- | --- | --- | --- | --- |
| **<1** | **0** | **56** | **1** | **SD** | **6** |
| **<1** | **1** | **40** | **1** | **SD** | **2** |
| **1-2** | **0** | **60** | **1** | **SD** | **5** |
| **1-2** | **1** | **63** | **0** | **SD** | **6** |
| **2-3** | **0** | **47** | **1** | **PR** | **3** |
| **2-3** | **1** | **58** | **0** | **PD** | **2** |
| **>3** | **0** | **26** | **1** | **PR** | **6** |
| **>3** | **1** | **58** | **0** | **PR** | **8** |

1. **Supplementary** **Figures**

**
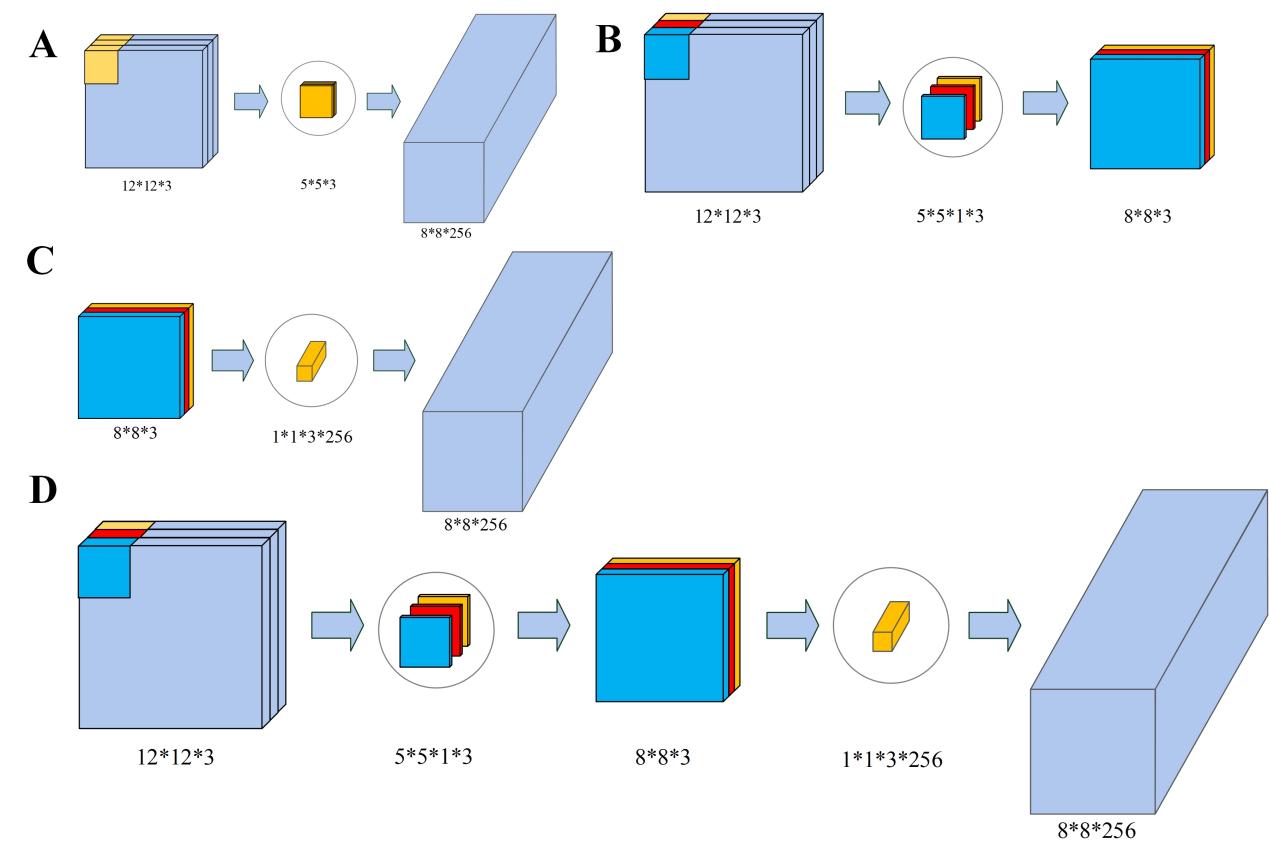
**

**sFigure 1** Detailed structures of different convolution
